# Supplementary material for: Biomimetic electromechanical stimulation to maintain adult myocardial slices in vitro
Source: Nat Commun. 2019 May 15;10:2168. doi: 10.1038/s41467-019-10175-3 (PMC6520377; doi:10.1038/s41467-019-10175-3)
Supplement: Supplementary file 1 — Supplementary Information [file 41467_2019_10175_MOESM1_ESM.pdf]

Biomimetic electromechanical stimulation to maintain adult myocardial slices in vitro

Watson et al.

Supplementary Information

Supplementary Figure 1

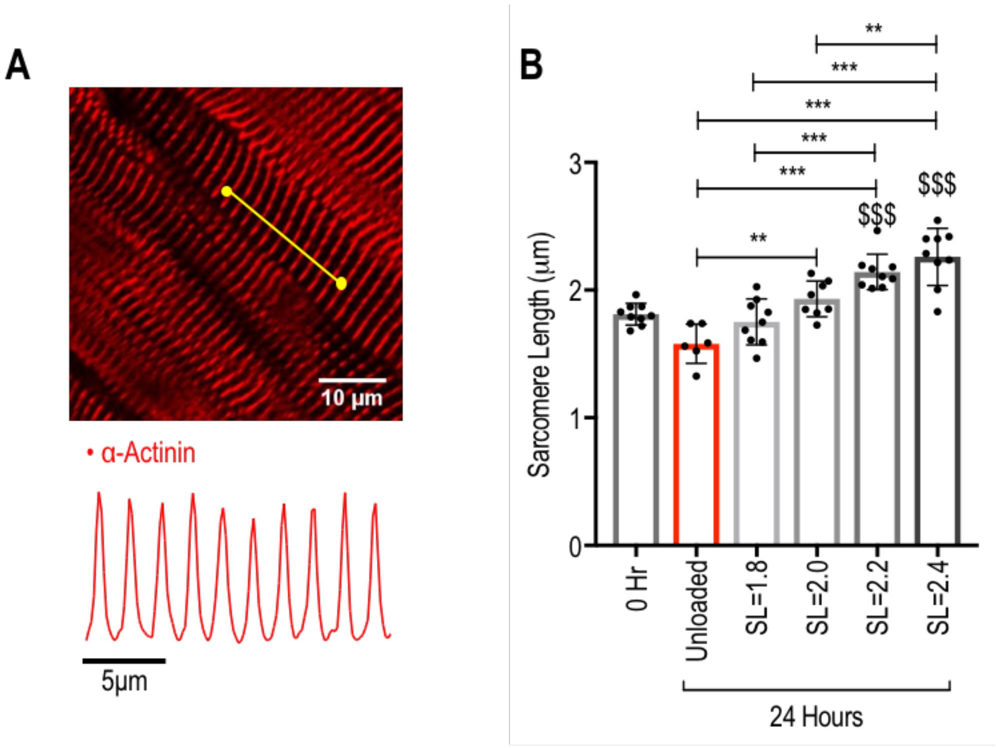

Immunohistochemical staining of α-Actinin to investigate rat myocardial slice sarcomere length

Immunohistochemical staining and confocal microscopy of fresh and cultured rat myocardial slices to investigate average slice sarcomere length. A) Top -Rat myocardial slice stained for α-Actinin. To measure average slice sarcomere length, the distance between 10 consecutive sarcomere was measured. This was repeated in 10 cells per slice and the average of the cells was used. Bottom – Profile plot of 10 consecutive sarcomere. Plot correlates with the yellow line in the image above. B) Assessment of average rat slice sarcomere length using immunohistochemical staining (α-actinin) and confocal microscopy (0 Hr, SL=1.8, 2.2 & 2.4 N=9, Unloaded N=6, SL=2.0 N=8). N = number of myocardial slices. Black dots represent individual data points. Mean ± standard error is shown on graphs. One-way analysis of variance (ANOVA) was used to determine whether there were any statistically significant differences between the means of groups. $, $$, $$$ = p value < 0.05, 0.01 & 0.001 respectively compared to 0Hr. *, **, *** = p value < 0.05, 0.01 & 0.001 respectively between the two groups highlighted by bar. Source data are provided as a Source Data file.

Supplementary Figure 2

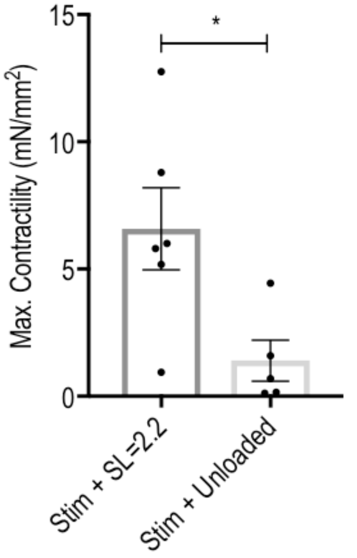

Mechanical load is required to culture myocardial slices

Rat myocardial slices were cultured on stretchers in culture chambers with 1Hz electrical stimulation (width: 10ms, voltage: 30V) for 24 hours. Slices were cultured with a preload equivalent to SL=2.2μm or unloaded (on stretcher with slice length < resting slice length). Unloaded slices had a significantly reduced contractility at 24 hours (Stim + SL=2.2 N=6, Stim + Unloaded N=5). N = number of myocardial slices. Black dots represent individual data points. Mean ± standard error is shown on graphs. An unpaired t-test was used to determine whether there were any statistically significant differences between the means of groups. * = p value < 0.05 between the two groups highlighted by bar. Source data are provided as a Source Data file.

Supplementary Figure 3

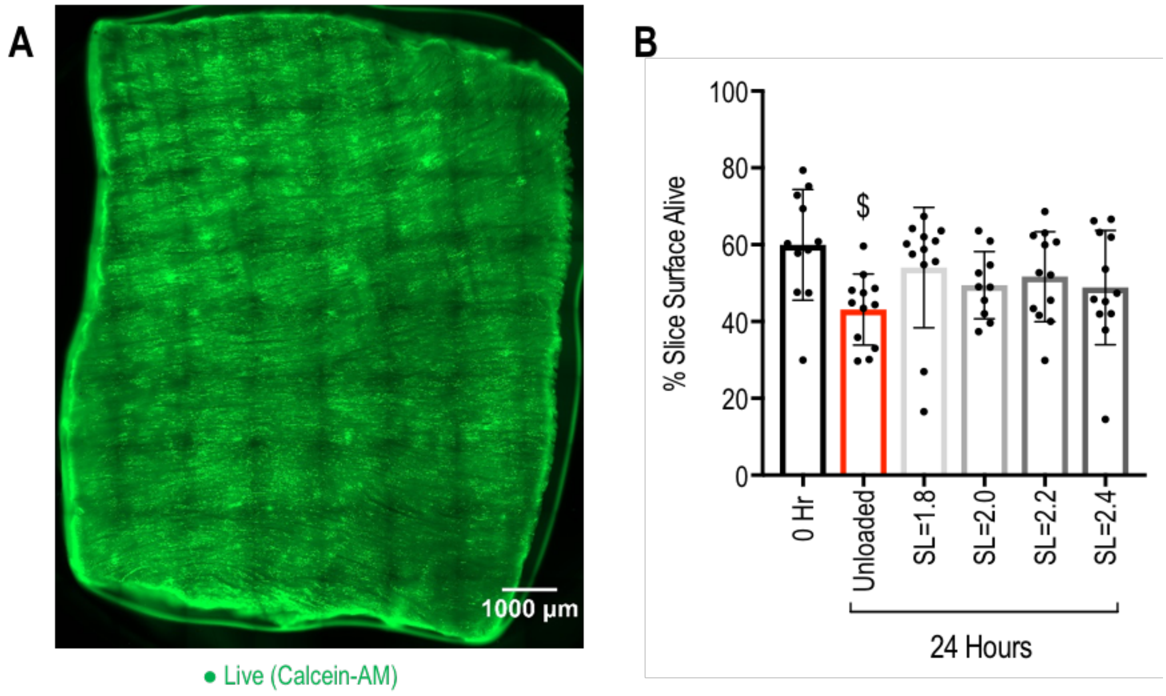

Myocardial slice surface Live/Dead viability assay

A) Whole 0Hr rat myocardial slice (including aligned and non-aligned areas) with the majority of the surface stained with calcein-AM (green). Tiled images of the whole myocardial slice were automatically collected using a x10 objective on a Zeiss AxioObserver equipped with a motorised stage. B) The % of the rat myocardial slice surface that was alive was quantified by measuring the percentage of the slice surface that was stained green using ImageJ. We found 60% of the surface of 0Hr slices were alive and that slices cultured with electromechanical stimulation and physiological level had a similar proportion of live cells on their surface at 24 hours. A small but significant reduction in cardiomyocyte viability was found on the surface of slices cultured in unloaded conditions. However, we have previously demonstrated that the layers of cardiomyocytes below the slice surface remain almost 100% viable[3] and the small reduction on the surface of unloaded slices would only account for ~1% of the total slice cardiomyocyte population (0 Hr N=11, Unloaded, SL=1.8, 2.2 & 2.4 N=12, SL=2.0 N=10). N = number of myocardial slices. Black dots represent individual data points. Mean ± standard error is shown on graphs. One-way analysis of variance (ANOVA) was used to determine whether there were any statistically significant differences between the means of groups. $, $$, $$$ = p value < 0.05, 0.01 & 0.001 respectively compared to 0Hr. *, **, *** = p value < 0.05, 0.01 & 0.001 respectively between the two groups highlighted by bar. Source data are provided as a Source Data file.

Supplementary Figure 4

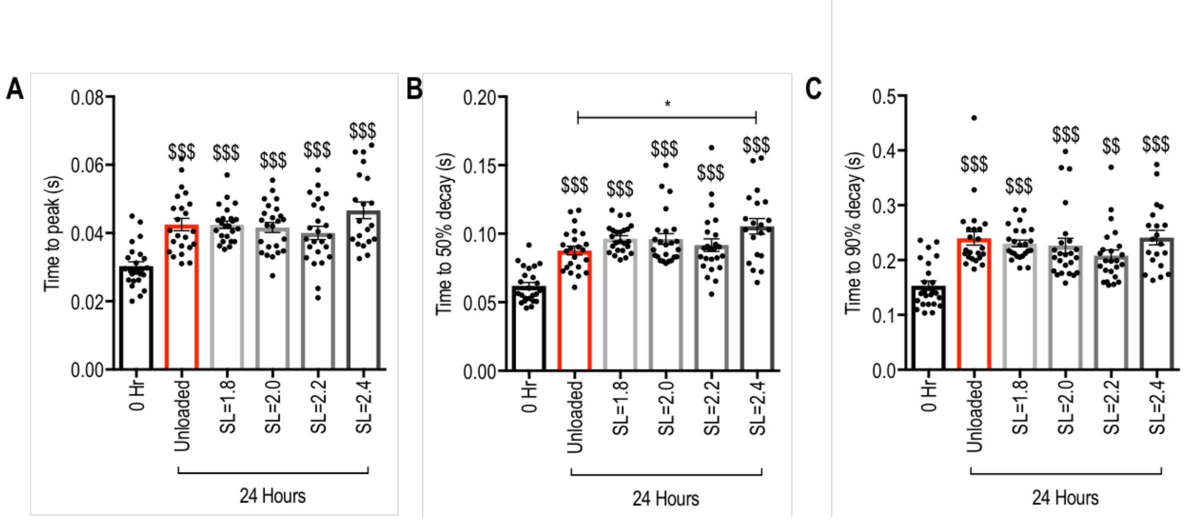

Ca^{2+} handling kinetics of rat myocardial slices

A) Time to peak of rat myocardial slices cultured for 24 hours (N=24/6, except SL=2.4 N=20/6) B) Time to 50% Ca^{2+} decay of rat myocardial slices cultured for 24 hours (N=24/6, except SL=2.4 N=20/6). C) Time to 90% Ca^{2+} decay of rat myocardial slices cultured for 24 hours (N=24/6, except SL=2.4 N=20/6). For Ca^{2+} handling data, N = number of regions analysed / number of myocardial slices. Black dots represent individual data points. Mean ± standard error is shown on graphs. One-way analysis of variance (ANOVA) was used to determine whether there were any statistically significant differences between the means of groups. $, $$, $$$ = p value < 0.05, 0.01 & 0.001 respectively compared to 0Hr. *, **, *** = p value < 0.05, 0.01 & 0.001 respectively between the two groups highlighted by bar. Source data are provided as a Source Data file.

Supplementary Figure 5

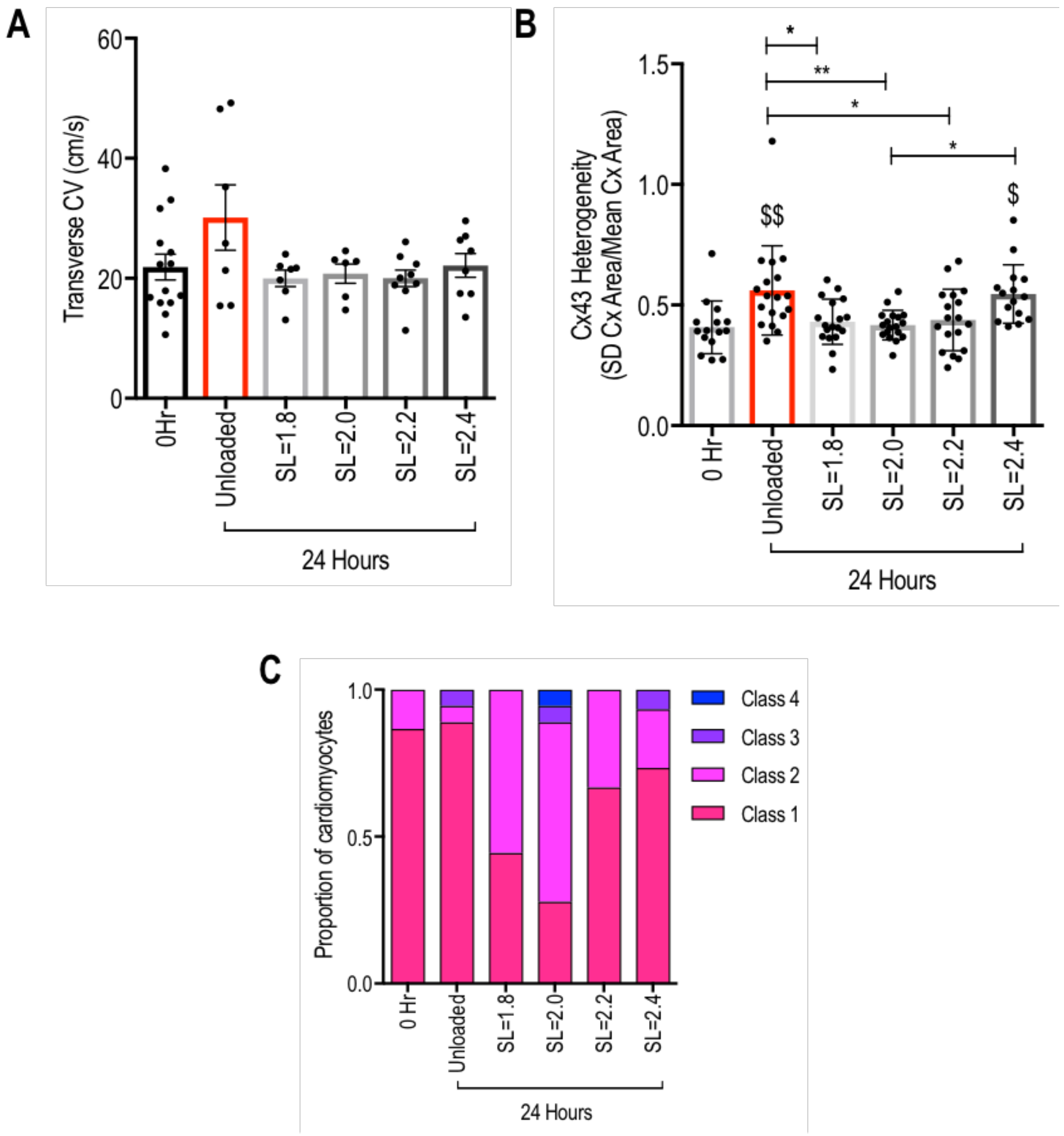

Connexin 43 expression heterogeneity of rat myocardial slices

A) Transverse conduction velocity, measured using point stimulation and a multielectrode array, of rat myocardial slices cultured for 24 hours (0 Hr N=14, Unloaded, SL=1.8 N=7, SL=2.0 N=6, SL=2.2 N=9, SL=2.4 N=8). B) Heterogeneity of connexin 43 expression is significantly increased with unloading and SL=2.4μm but preserved at SL=1.8-2.2μm (N=18/6, except 0 Hr & SL=2.4 N=15/6). To assess heterogeneity of Cx43 density within the slices, Z-stacks of 1400x1400μm images were acquired and a minimum threshold for Cx43 signal of 50 applied, as previously described[50] The area of Cx43 signal was measured in 700x700□m quadrants to correlate with the distance between multielectrode array electrodes. The standard deviation of values for Cx43 density in quadrants was calculated as a measure of heterogeneity within each image, as previously described[51] These values were corrected to the mean Cx43 density of each image to allow for comparison of relative heterogeneity between culture conditions with differing overall Cx43 density C) Cx43 lateralisation was investigated using a semi-quantitative technique as previously described[52]. No differences in lateralisation patterns were found between any condition (N=18/6). For conduction velocity, N = number of myocardial slices. Black dots represent individual data points. Mean ± standard error is shown on graphs. For Cx43 lateralisation, N = number of area analysed / number of myocardial slices. One-way analysis of variance (ANOVA) was used to determine whether there were any statistically significant differences between the means of groups. $, $$, $$$ = p value < 0.05, 0.01 & 0.001 respectively compared to 0Hr. *, **, *** = p value < 0.05, 0.01 & 0.001 respectively between the two groups highlighted by bar. Source data are provided as a Source Data file.

Supplementary Figure 6

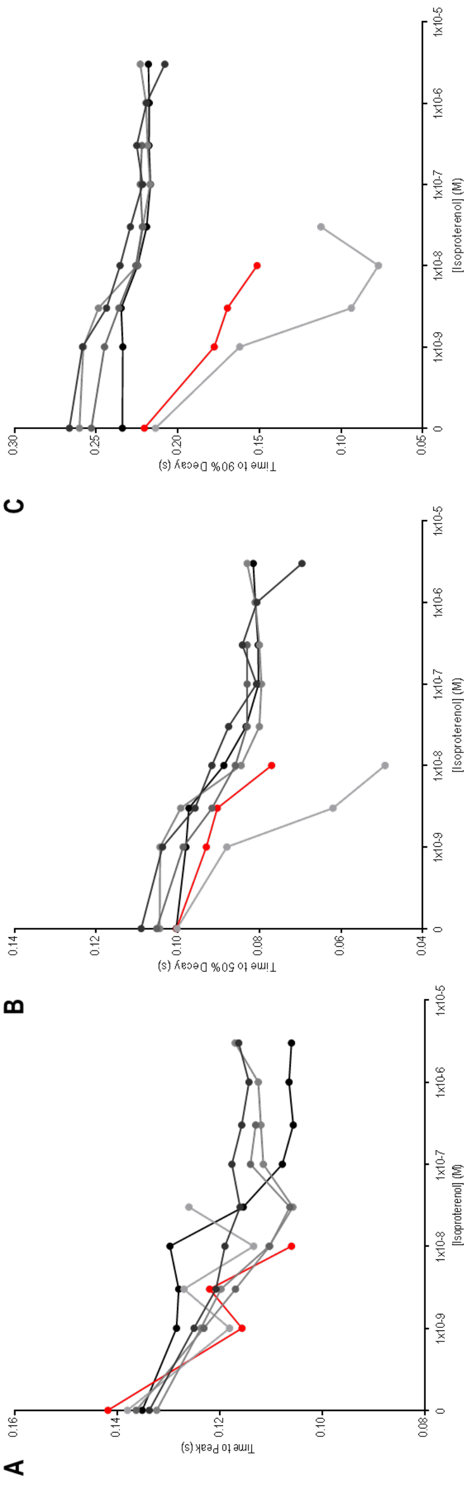

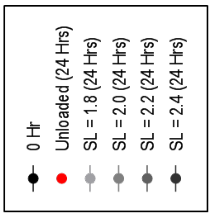

D

	Force	
	EC_{50} (M)	95% CI
0Hr	1.16×10^{-8}	4.15×10^{-9} to 3.23×10^{-8}
Unloaded	NA	NA
SL=1.8	NA	NA
SL=2.0	1.80×10^{-8}	5.14×10^{-9} to 6.30×10^{-8}
SL=2.2	4.89×10^{-9}	2.04×10^{-9} to 1.18×10^{-8}
SL=2.4	1.43×10^{-8}	4.79×10^{-9} to 4.26×10^{-8}

Rat myocardial slice contraction kinetics with isoproterenol stimulation & isoproterenol EC_{50} for contractile force

A) Time to peak force of rat myocardial slices cultured for 24 hours and then stimulated with increasing concentrations of isoproterenol (0Hr & SL=2.0-2.4μm - N=7, unloaded – N=4, SL=1.8μm – N=5). B) Time to 50% force decay of rat myocardial slices cultured for 24 hours and then stimulated with increasing concentrations of isoproterenol (0Hr & SL=2.0-2.4μm - N=7, unloaded – N=4, SL=1.8μm – N=5). C) Time to 90% force decay of rat myocardial slices cultured for 24 hours and then stimulated with increasing concentrations of isoproterenol (0Hr & SL=2.0-2.4μm - N=7, unloaded – N=4, SL=1.8μm – N=5). D) Isoproterenol concentration EC_{50} and confidence intervals for contractile force of rat myocardial slices cultured for 24 hours and then stimulated with increasing concentrations of isoproterenol (0Hr & SL=2.0-2.4μm - N=7, unloaded – N=4, SL=1.8μm – N=5). N = number of myocardial slices. Mean is shown on graphs (A-C). Source data are provided as a Source Data file.

Supplementary Figure 7

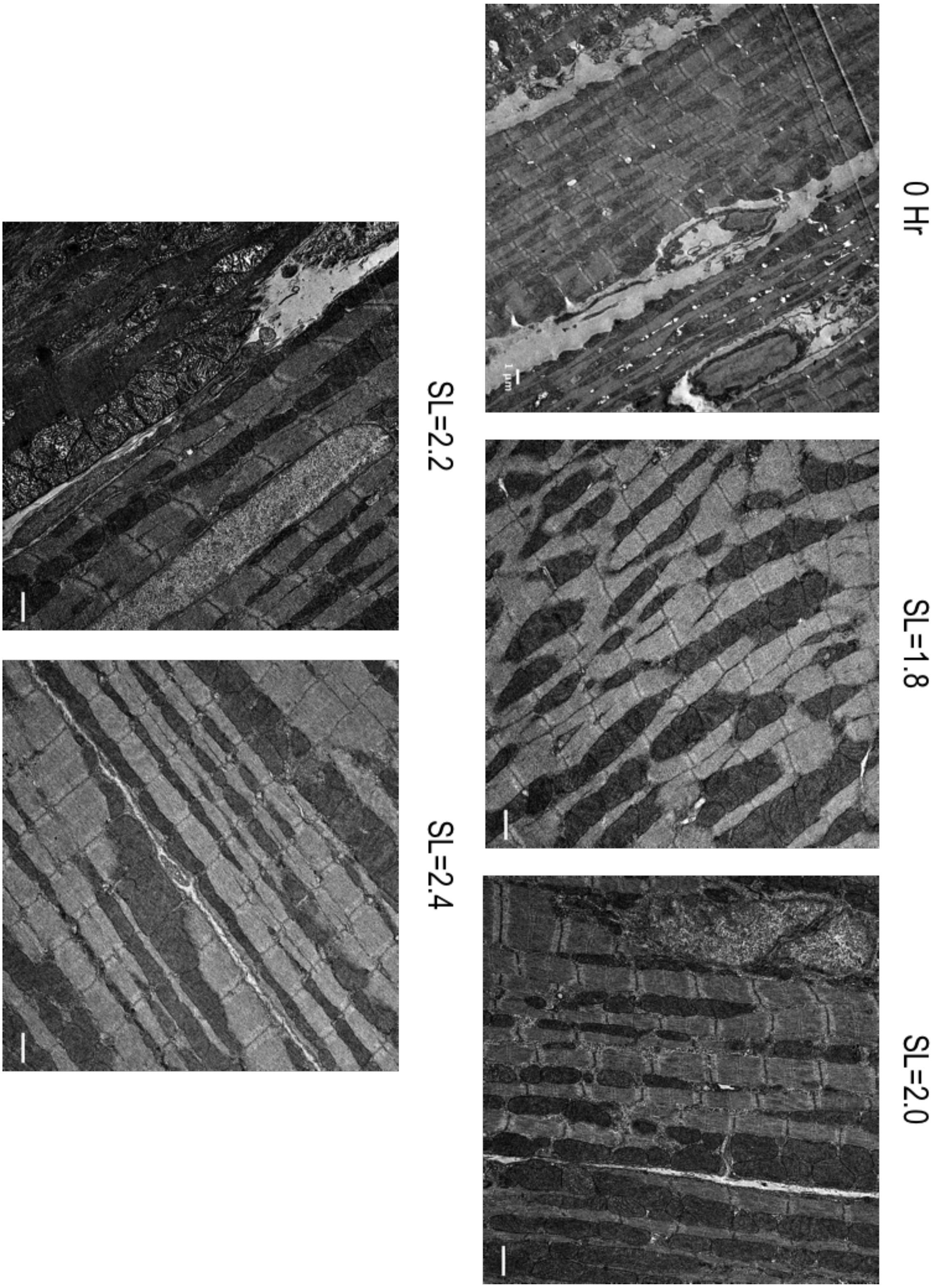

Transmission electron microscopy of rat myocardial slices

Transmission electron microscopy was performed to assess the structural properties of rat myocardial slices cultured with electromechanical stimulation for 24 hours. At all SLs, both the architecture of the myocardium and the ultrastructure of cardiomyocytes were preserved. Scale bar = 1μm.

Supplementary Figure 8

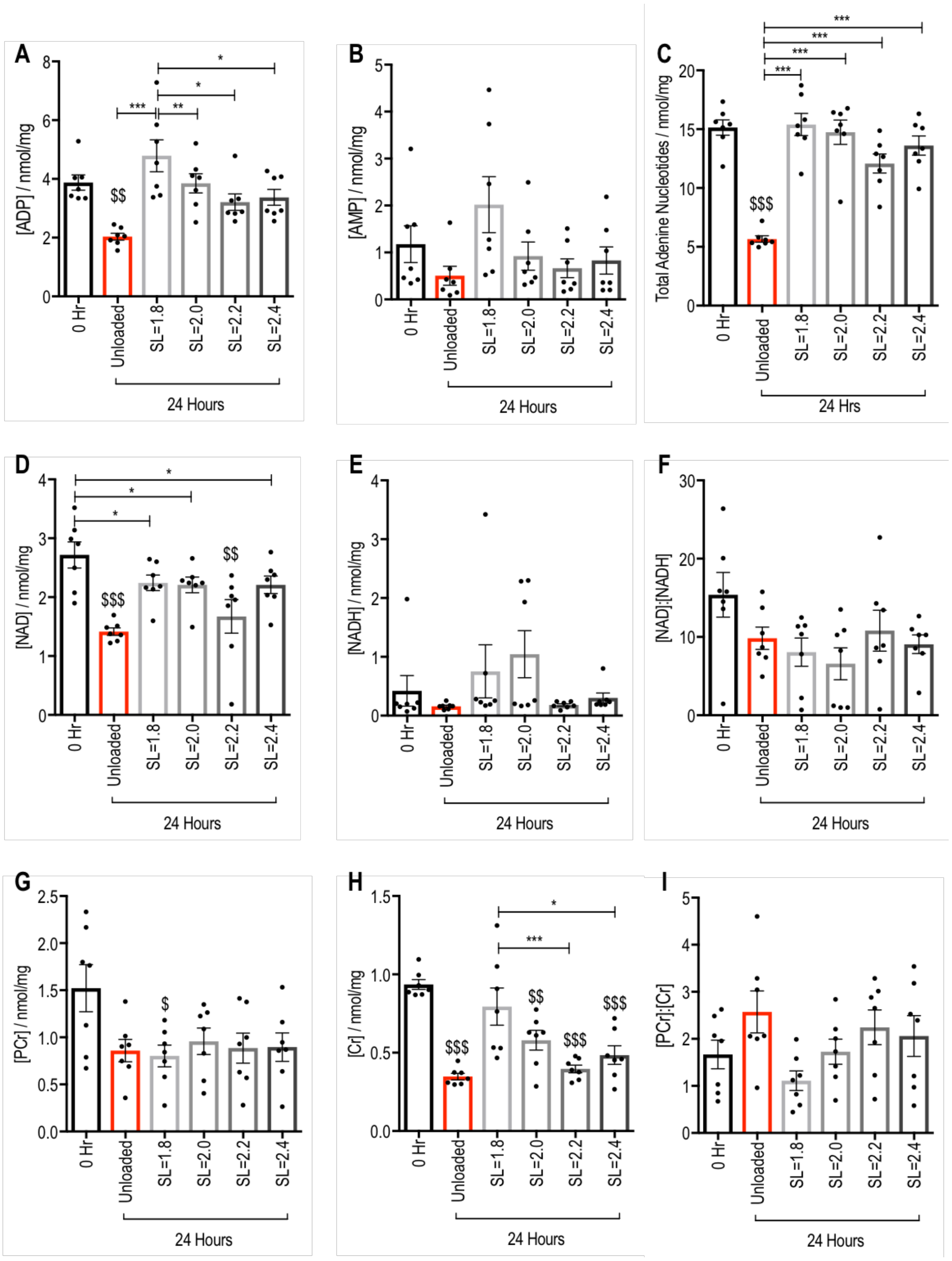

Myocardial slice metabolite concentrations measured with high performance liquid chromatography

A) [ADP] in rat myocardial slices cultured for 24 hours (N=7). B) [AMP] in rat myocardial slices cultured for 24 hours (N=7). C) Total adenine nucleotides in rat myocardial slices cultured for 24 hours (N=7). D) [NAD] in rat myocardial slices cultured for 24 hours (N=7). E) [NADH] in rat myocardial slices cultured for 24 hours (N=7). F) [NAD]:[NADH] in rat myocardial slices cultured for 24 hours (N=7). G) [PCr] in rat myocardial slices cultured for 24 hours (N=7). H) [Cr] in rat myocardial slices cultured for 24 hours (N=7). I) [PCr]:[Cr] in rat myocardial slices cultured for 24 hours (N=7). N = number of myocardial slices. Black dots represent individual data points. Mean ± standard error is shown on graphs. One-way analysis of variance (ANOVA) was used to determine whether there were any statistically significant differences between the means of groups. $, $$, $$$ = p value < 0.05, 0.01 & 0.001 respectively compared to 0Hr. *, **, *** = p value < 0.05, 0.01 & 0.001 respectively between the two groups highlighted by bar. Source data are provided as a Source Data file.

Supplementary Figure 9

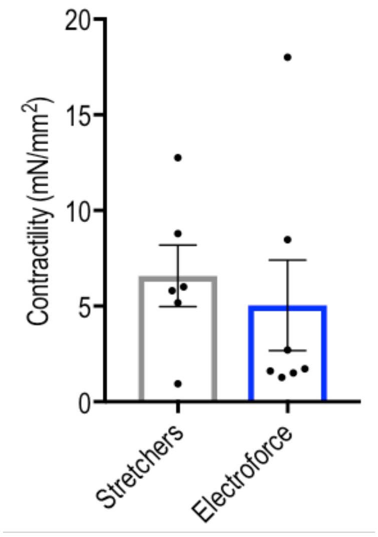

Myocardial slice stretchers and the Bose Electroforce set up provide comparable outcomes at 24 hours

Rat myocardial slices cultured at SL=2.2μm for 24 hours using myocardial slice stretchers and the Bose Electroforce set up (Stretcher - N=6, Electroforce – N=7). N = number of myocardial slices. Black dots represent individual data points. Mean ± standard error is shown on graphs. An unpaired T-test was used to determine whether there were any statistically significant differences between the means of groups. No statistical difference was found. Source data are provided as a Source Data file.
