# Supplementary material for: Biomimetic electromechanical stimulation to maintain adult myocardial slices in vitro
Source: Nat Commun. 2019 May 15;10:2168. doi: 10.1038/s41467-019-10175-3 (PMC6520377; doi:10.1038/s41467-019-10175-3)
Supplement: Supplementary file 3 — Reporting Summary [file 41467_2019_10175_MOESM3_ESM.pdf]

nature research

Corresponding author(s): Cesare Terracciano

Last updated by author(s): Mar 30, 2019

Reporting Summary

Nature Research wishes to improve the reproducibility of the work that we publish. This form provides structure for consistency and transparency in reporting. For further information on Nature Research policies, see Authors & Referees and the Editorial Policy Checklist.

Statistics

For all statistical analyses, confirm that the following items are present in the figure legend, table legend, main text, or Methods section.

n/a	Confirmed	
☐	☒	The exact sample size (*n*) for each experimental group/condition, given as a discrete number and unit of measurement
☐	☒	A statement on whether measurements were taken from distinct samples or whether the same sample was measured repeatedly
☐	☒	The statistical test(s) used AND whether they are one- or two-sided<br>*Only common tests should be described solely by name; describe more complex techniques in the Methods section.*
☒	☐	A description of all covariates tested
☐	☒	A description of any assumptions or corrections, such as tests of normality and adjustment for multiple comparisons
☐	☒	A full description of the statistical parameters including central tendency (e.g. means) or other basic estimates (e.g. regression coefficient) AND variation (e.g. standard deviation) or associated estimates of uncertainty (e.g. confidence intervals)
☐	☒	For null hypothesis testing, the test statistic (e.g. *F*, *t*, *r*) with confidence intervals, effect sizes, degrees of freedom and *P* value noted<br>*Give P values as exact values whenever suitable.*
☒	☐	For Bayesian analysis, information on the choice of priors and Markov chain Monte Carlo settings
☒	☐	For hierarchical and complex designs, identification of the appropriate level for tests and full reporting of outcomes
☒	☐	Estimates of effect sizes (e.g. Cohen's *d*, Pearson's *r*), indicating how they were calculated

Our web collection on statistics for biologists contains articles on many of the points above.

Software and code

Policy information about availability of computer code

Data collection	Axoscope software, Molecular Devices, USA<br>HCImage Live software, Hamamatsu, Japan<br>MC_Rack, Multichannel Systems, Germany<br>LT-com PC Control Software, Labtech, UK<br>Zen, Zeiss, Germany
Data analysis	Clampfit software, Molecular Devices, USA<br>Prism 7, GraphPad, USA<br>MATLAB, Mathworks, USA<br>GSEA software, Broad Institute<br>Excel, Microsoft, USA

For manuscripts utilizing custom algorithms or software that are central to the research but not yet described in published literature, software must be made available to editors/reviewers. We strongly encourage code deposition in a community repository (e.g. GitHub). See the Nature Research guidelines for submitting code & software for further information.

Data

Policy information about availability of data

All manuscripts must include a data availability statement. This statement should provide the following information, where applicable:

- Accession codes, unique identifiers, or web links for publicly available datasets
- A list of figures that have associated raw data
- A description of any restrictions on data availability

Source data for figures 1B, 2B-C, 2E-G, 2I-M, 3C-D, 3F-J, 4B-C, 4E-F, 4H-J, 5A-B, 6A-B and supplementary figures 1B, 2, 3, 4A-C, 5A-C, 6A-C, 8A-I, 9 are provided with the paper. RNA-seq data have been deposited in the ArrayExpress database at EMBL-EBI (www.ebi.ac.uk/arrayexpress) under accession number E-MTAB-7842

[https://www.ebi.ac.uk/arrayexpress/experiments/E-MTAB-7842]. Full microscopy image data sets are available from the corresponding author upon reasonable request.

Field-specific reporting

Please select the one below that is the best fit for your research. If you are not sure, read the appropriate sections before making your selection.

☒ Life sciences ☐ Behavioural & social sciences ☐ Ecological, evolutionary & environmental sciences

For a reference copy of the document with all sections, see nature.com/documents/nr-reporting-summary-flat.pdf

Life sciences study design

All studies must disclose on these points even when the disclosure is negative.

Sample size	A sample size greater than or equal to 6 was collected for each experiment. When this was not possible, this is clearly stated and all raw data is represented as dots in figures. A sample size greater than or equal to 6 was sufficient to identify statistically significant differences between groups.
Data exclusions	No data were excluded.
Replication	The findings detailed in this paper have been replicated several times within our laboratory. The data was collected by several individuals.
Randomization	All samples were randomly assigned electromechanical stimulation protocol.
Blinding	Groups were blinded during data collection and analysis whenever possible.

Reporting for specific materials, systems and methods

We require information from authors about some types of materials, experimental systems and methods used in many studies. Here, indicate whether each material, system or method listed is relevant to your study. If you are not sure if a list item applies to your research, read the appropriate section before selecting a response.

Materials & experimental systems

n/a	Involved in the study	
☐	☒	Antibodies
☒	☐	Eukaryotic cell lines
☒	☐	Palaeontology
☐	☒	Animals and other organisms
☐	☒	Human research participants
☒	☐	Clinical data

Methods

n/a	Involved in the study	
☒	☐	ChIP-seq
☒	☐	Flow cytometry
☒	☐	MRI-based neuroimaging

Antibodies

Antibodies used

Antibody / Stain How it was created / Dilution / Manufacturer / Catalogue Number

Primary Anitbodies
Caveolin 3 / Raised in Mouse / 1 : 500 / BD Biosciences / 610421
Connexin 43 / Raised in Rabbit / 1 : 2000 / Sigma-Aldrich / C6219
a-Actinin / Raised in Mouse / 1 : 2000 / Sigma-Aldrich / A7811
Vimentin / Raised in Chicken / 1 : 3000 / Thermo Fisher Scientific / PA1-16759
TOM20 / Raised in Mouse / 1 : 750 / Santa-Cruz / sc-17764
Isolectin B4 / Biotin-conjugated / 1 : 1000 / Life Technologies / I21414

Secondary Antibodies
Alexa 488 / Raised in Donkey, Anti-Mouse / 1 : 2000 / Life Technologies / A21202
Alexa 546 / Raised in Donkey, Anti-Rabbit / 1 : 2000 / Life Technologies / A10040
Alexa 647 / Raised in Goat, Anti-Chicken / 1 : 2000 / Life Technologies / A-21449

Nuclear Staining
Hoechst 33342 / 1 : 1000 / Life Technologies / H3570

Validation

All antibodies have been validated within our laboratory for previously published work.

Nat Protoc. 2017 Dec;12(12):2623-2639. doi: 10.1038/nprot.2017.139. Epub 2017 Nov 30.

Cardiovasc Res. 2018 Jan 1;114(1):77-89. doi: 10.1093/cvr/cvx152.
Sci Rep. 2017 Jul 12;7(1):5188. doi: 10.1038/s41598-017-05406-w.

Animals and other organisms

Policy information about studies involving animals; ARRIVE guidelines recommended for reporting animal research

Laboratory animals	Rat, Sprague Dawley, Male, 250-500g<br>Rabbit, New Zealand White, Male, 2.5-3.0Kg
Wild animals	The study did not involve wild animals
Field-collected samples	The study did not involve samples collected from the field.
Ethics oversight	All animal experiments complied with Institutional and National regulations. Our use of living cardiac tissue was approved by Imperial College London. The procedures we describe here were performed under license by the UK Home Office, in accordance with the United Kingdom Animals (Scientific Procedures) Act 1986. Animals were killed following guidelines established by the European Directive on the protection of animals used for scientific purposes (2010/63/EU).

Note that full information on the approval of the study protocol must also be provided in the manuscript.

Human research participants

Policy information about studies involving human research participants

Population characteristics	All patients had end-stage heart failure (secondary to dilated cardiomyopathy) and were undergoing cardiac transplantation.
Recruitment	All patients agreed to donate tissue to the study prior to cardiac transplantation and provided informed consent.
Ethics oversight	Human HF samples were provided by the NIHR Cardiovascular Biomedical Research Unit at the Royal Brompton and Harefield NHS Foundation Trust and Imperial College London. The study performed conformed to the principles outlined in the Declaration of Helsinki and the investigation was approved by a UK institutional ethics committee (NRES ethics number for biobank samples: 09/H0504/104+5; Biobank approval number: NP001-06-2015 & MED_CT_17_079) & Imperial College London. Informed consent was obtained from each patient involved in this study.

Note that full information on the approval of the study protocol must also be provided in the manuscript.
